# Prevalence of tuberculosis in HIV in Ethiopia in early HAART era: retrospective analysis

**DOI:** 10.11604/pamj.2013.14.126.1907

**Published:** 2013-04-01

**Authors:** Esayas Kebede Gudina, Fikadu Girma Gudissa

**Affiliations:** 1 Jimma University, Jimma, Ethiopia

**Keywords:** Tuberculosis, HAART, Ethiopia, HIV

## To the editors of the Pan African Medical Journal

The intersection of the human immunodeficiency virus (HIV) and tuberculosis (TB) epidemics has led to a dramatic upsurge in global TB incidence, resulting in remarkable increases in morbidity and mortality [1–6]. In Ethiopia where the burden of both diseases is immense [7, 8] and where access to highly active antiretroviral therapy (HAART) was once limited, we found it useful to share our findings about TB/HIV interaction during early HAART era; our findings could serve as a base line against which the future impact of HAART would be assessed.

We did a retrospective review of charts of 287 patients on follow-up at ART clinic of Jimma University Hospital during the early HAART era (between 2003 and 2006). It was found that 57% of the participants were females and 78% belonged to the age group 20 to 40 years.

More than half of the patients presented with at least one symptoms of chronic unexplained fever, chronic diarrhea, chronic cough or oropharyngeal candidiasis at the time of HIV diagnosis. Seventy-seven percent had advanced disease (WHO stages 3 or 4) at diagnosis. Only 73% had their CD4 count done at the time of HIV diagnosis, 36% of them had <200 cells/µL (Table 1).

**Table 1:** Clinical conditions of HIV patients at the time of diagnosis at ART clinic, JUSH from June 2003- July 7, 2006

Characteristics	Frequency	%
**Signs and symptoms**		
Chronic unexplained fever	166	57.8
Chronic diarrhea	142	49.5
Chronic cough	110	38.3
Oropharyngeal candidiasis	113	39.4
HIV wasting syndrome	82	24.4
Extrapulmonay tuberculosis	24	8.4
Generalized lymphadenopathy	8	2.9
Toxoplasmosis	7	2.4
No sign and symptom	13	4.5
Others	33	11.5
**WHO staging**		
Stage 1	4	1.4
Stage 2	62	21.6
Stage 3	130	45.3
Stage 4	91	31.7
**CD_4_ count (cells/L)**		
<50	19	6.3
50-99	34	11.8
100-199	51	17.8
200-499	71	24.7
500+	35	12.2
Not done	77	26.8

Forty-five percent of the patients had HIV associated tuberculosis considered as clinical TB dating from two years prior to HIV diagnosis and ever since. Sixteen percent had active TB at time of HIV diagnosis or developed it since then. Of those diagnosed with active TB, 44% had previous history of TB. Only about 20% of them had smear-positive TB and thus diagnosis was based on constitutional symptoms or abnormal chest X-ray findings. Over half of them had isolated pulmonary TB and 41% had the disseminated form of the disease involving two or more organ systems. Lower CD4 counts and advanced WHO stages of the disease at time of diagnosis were strong predictors for the occurrence of active TB (p < 0.01) (Table 2).

**Table 2:** Predictors of new active TB at and after diagnosis of HIV at ART clinic, JUSH from June 2003- July 7, 2006

Characteristics	Presence of active TB		P-value
	Yes, N (%)	No, N (%)	
**Previous TB**			>0.2
Yes	20 (19.6)	82 (80.4)	
No	26 (14.1)	159 (85.9)	
**CD_4_ count**			<0.01
<200	27 (26.0)	77 (74.0)	
200-499	5 (7.0)	66 (93.0)	
500+	2 (5.7)	33 (94.3)	
unknown	12 (15.6)	65 (84.4)	
**WHO stage**			<0.01
1	0 (0)	4 (100)	
2	5 (8.1)	57 (91.9)	
3	15 (11.5)	115 (88.5)	
4	26 (28.6)	65 (71.4)	

In conclusion, TB morbidity in HIV patients was found to be rampant in Ethiopia, in a setting where access for HAART was poor. The high proportion of disseminated forms of TB, atypical diagnostic findings and patients presentation at the advanced stage of the HIV were found to be further setback in such resource stretched country. The findings of this study will give additional information regarding the burden of TB in HIV patients in Ethiopia where there are only few published studies in the field [8]. It has focused on burden of TB in early HAART era and will importantly lay ground for further studies to be done to see whether an increased access for ART has decreased TB incidence in HIV patients. We however strongly recommend a large scale multicenter prospective study to see the real TB/HIV comorbidity, the course of TB in HIV patients and the effect of HAART on TB prevalence.
